# Trackplot: A flexible toolkit for combinatorial analysis of genomic data

**DOI:** 10.1371/journal.pcbi.1011477

**Published:** 2023-09-05

**Authors:** Yiming Zhang, Ran Zhou, Lunxu Liu, Lu Chen, Yuan Wang

**Affiliations:** 1 Department of Neurosurgery and State Key Laboratory of Biotherapy and Cancer Center, West China Hospital, Sichuan University, Chengdu, Sichuan, China; 2 Institute of Thoracic Oncology and Department of Thoracic Surgery, West China Hospital, Sichuan University, Chengdu, Sichuan, China; 3 State Key Laboratory of Biotherapy, West China Second University Hospital, Sichuan University, Chengdu, Sichuan, China; Burnet Institute, AUSTRALIA

## Abstract

Here, we introduce Trackplot, a Python package for generating publication-quality visualization by a programmable and interactive web-based approach. Compared to the existing versions of programs generating sashimi plots, Trackplot offers a versatile platform for visually interpreting genomic data from a wide variety of sources, including gene annotation with functional domain mapping, isoform expression, isoform structures identified by scRNA-seq and long-read sequencing, as well as chromatin accessibility and architecture without any preprocessing, and also offers a broad degree of flexibility for formats of output files that satisfy the requirements of major journals. The Trackplot package is an open-source software which is freely available on Bioconda (https://anaconda.org/bioconda/trackplot), Docker (https://hub.docker.com/r/ygidtu/trackplot), PyPI (https://pypi.org/project/trackplot/) and GitHub (https://github.com/ygidtu/trackplot), and a built-in web server for local deployment is also provided.

## Author summary

Simultaneously visualizing how isoform expression, protein-DNA/RNA interactions, accessibility, and architecture of chromatin differs across conditions and cell types could inform our understanding on regulatory mechanisms and functional consequences of alternative splicing. However, the existing versions of tools generating sashimi plots remain inflexible, complicated, and user-unfriendly for integrating data sources from multiple bioinformatic formats or various genomics assays. Thus, a more scalable visualization tool is necessary to broaden the scope of sashimi plots. To overcome these limitations, we present Trackplot, a comprehensive tool that delivers high-quality plots via a programmable and interactive web-based platform. Trackplot seamlessly integrates diverse data sources and utilizes a multi-threaded process, enabling users to explore genomic signal in large-scale sequencing datasets.

## Introduction

Uncovering differential isoform expression is crucial for enhancing proteome diversity and transcript functionality [1]. Various library protocols and sequencing methods, such as single-cell RNA sequencing (scRNA-seq) [2] and long-read sequencing [3], have been developed and widely used to explore the heterogeneity of isoform expression in single cells. Despite the availability of advanced tools for analyzing and visualizing genomics data, several challenges persist. Existing tools like sashimi [4], ggsashimi [5], and SplicePlot [6] are limited in efficiency and flexibility when handling the ever-growing volume and size of data. Moreover, these tools often only provide a command-line interface, which can be daunting for inexperienced programmers. Additionally, conventional interactive genome browsers like Integrative Genomics Viewer (IGV) [7] lack flexibility in output format. To address these limitations, we introduce Trackplot, a comprehensive tool that generates high-quality plots in a programmable and interactive web-based format. Trackplot offers integrated visualization of diverse data sources, including gene annotation with functional domain mapping, isoform expression, isoform structures identified by scRNA-seq and long-read sequencing, as well as chromatin accessibility and architecture.

### Design and implementation

**Fig 1:**
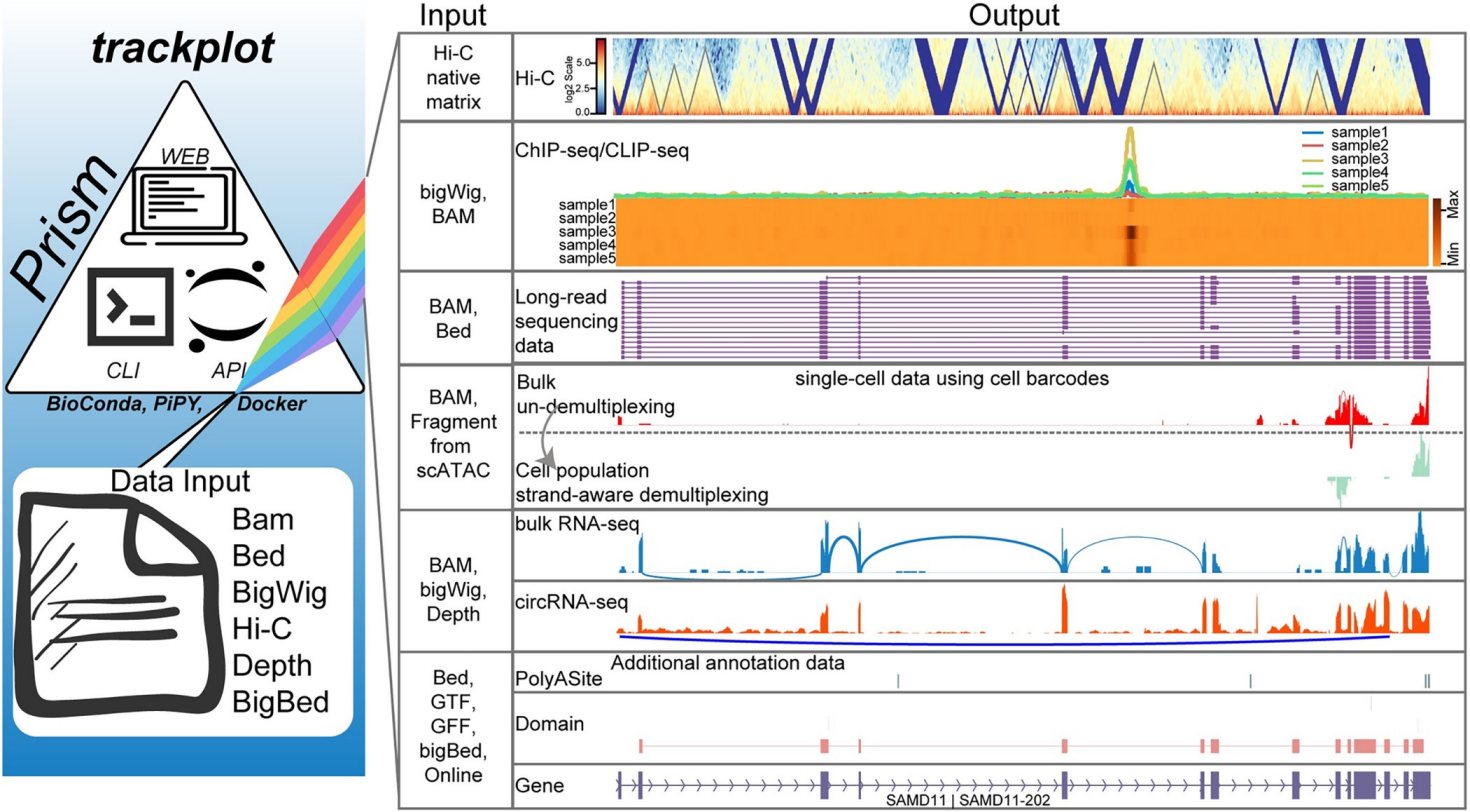
The diagram illustrates the architecture of Trackplot. It is developed using python and JavaScript which greatly facilitates future maintenance, could visualize genomic data from a variety of sources and bioinformatic formats on the given genomic coordinates.

Trackplot is a platform that leverages Python and JavaScript to visualize genomic data from diverse sources and generate plots suitable for publication. It offers easy accessibility and ensures high reproducibility. Users can freely download Trackplot from GitHub and install it from source code, PyPI, Pipenv, Bioconda, AppImage, or a Docker image. It provides multiple approaches for generating plots, including an application programming interface (API) for scripts and Jupyter Notebooks, a command-line interface (CLI), and a user-friendly web interface. Trackplot supports most standard data formats in bioinformatics, such as BAM, BED, bigWig, bigBed, GTF, BedGraph, HiCExplorer’s native h5 format, and the depth file generated by samtools [8] (Fig 1).

To generate plots, Trackplot initially requires the precise genomic coordinates of interest and a meta file containing information such as file path, data category, display label, color, and strandness for each data track. If there is a need for additional annotations, such as cell meta for demultiplexing or highlight regions for polyadenylation sites, these can be provided as parameters. Additionally, when the domain model is activated, Trackplot incorporates an automated process to request the API (https://rest.uniprot.org/uniprotkb/search?&query="ID") of UniProt [9] using the transcript ID in order to retrieve its corresponding protein ID, which may have multiple values. Subsequently, each protein ID associated with the given transcript ID is utilized to access the ENSEMBL [10] API (https://www.ebi.ac.uk/proteins/api/features/"uniprot_ID"). The tool verifies whether the length of the coding sequence (CDS) is three times that of the protein length, and if so, it collects and visualizes the domain information with user-defined filters. Subsequently, all the configuration information will be stored in a Plot object for further processing. Once the configuration process is complete, the tool utilizes packages such as pysam, pyBigWig, or hicmatrix to parse the input track files. It extracts and stores comprehensive information, including abundance, splicing junctions, gene annotation, and protein domains of the target region, in a Pandas dataframe. This dataframe can then be utilized for further analysis and processing. Finally, Trackplot utilizes the matplotlib package to generate plots, which provides flexibility in adjusting the size and resolution (dots per inch, DPI) of the figure. It also supports various output formats, including *png*, *pdf*, and *tiff*, ensuring compatibility with the requirements of major scientific journals. In addition to supporting some features already present in existing software, such as sample aggregation, Reads per kilobase of transcript per Million reads mapped (RPKM) / reads per million (RPM) calculation, and intron shrinkage, our tool outperforms the existing sashimi tool in terms of speed and efficiency (S1 Fig). In summary, Trackplot provides a highly accessible, reproducible, and flexible tool for generating genomic data plots.

## Results

Trackplot functions similarly to previous Sashimi plot packages, taking all splicing reads including novel junctions from BAM files and gene model annotations from GTF or BED files as input to visualize the differential usage of exons or transcripts. An example of a plot generated by Trackplot for eight bulk RNA-seq samples from the TNP GBM model [11] is shown in S1A Fig, which suggests gradual exclusion of the middle exon during tumorigenesis. The tool identified that the long isoform, which encodes a protein with key functional domains, is gradually spliced out, and the short isoform without functional domains becomes the major isoform (S1A Fig). Moreover, trackplot could take input in various bioinformatics formats, making it flexible in integrating data from multiple sources. Through the integration of RNA binding signal data (bigWig) and coverage data (BAM), Trackplot effectively illustrates the enrichment of PTBP1 at exon 2 of *PTBP3*. This observation suggests that PTBP1 is likely to directly regulate the alternative splicing of *PTBP3*’s exon 2, consistent with previous findings [12] (S2B Fig).

The advent of long-read sequencing platforms, such as Pacific Biosciences and Oxford Nanopore Technologies, has revolutionized transcriptome analysis by providing full transcript structures without the need for assembly. However, existing sashimi plot tools are primarily designed for short-read sequencing data and visualize sequencing reads by aggregating the depth of each coordinate, thereby losing the exon connections from individual reads. This limitation is effectively addressed by Trackplot, which offers a read-by-read style visualization with exon-sort options. This unique feature enables Trackplot to distinctly present the exon-intron structures of each isoform, providing a more comprehensive view of the transcriptome (S3A Fig). Moreover, Trackplot has the capability to extract and visualize additional information from the BAM file tags, such as the length of poly(A) tails or the modification status of each nucleobase (S3B Fig). By incorporating these features, Trackplot offers enhanced insights into the complexity and diversity of transcriptomes.

Several methods have recently been proposed to identify and estimate alternative polyadenylation (APA) events at the single-cell level, including SCAPE [13]. Existing tools in the field lack the capability to accurately demultiplex gene expression into distinct cell populations, often requiring users to manually split and deduplicate BAM files prior to analysis. However, Trackplot offers an automated solution to this challenge by implementing a demultiplexing and deduplication process based on a user-provided meta file containing cell barcodes and their corresponding cell types. This feature enables Trackplot to generate a clearer and more accurate representation of differential expression APA (alternative polyadenylation) events among 3’ enriched single-cell RNA sequencing (scRNA-seq) data, as illustrated in S4A Fig. Furthermore, Trackplot extends its functionality to support the analysis of single-cell data that simultaneously profiles the transcriptome and chromatin accessibility. In an example analysis, Trackplot presents a differential chromatin accessibility pattern of *U2AF1L4* between CD4 naïve T cells and CD16 monocytes. This observation correlates with distinct usage patterns of alternative polyadenylation sites (pA1 and pA2) in these two cell populations, as depicted in S4B Fig. These findings highlight the utility of Trackplot in exploring the relationship between transcriptional enhancers and 3’ end processing. In summary, Trackplot provides a comprehensive platform for researchers to investigate isoform diversity within cell populations and explore potential enhancer elements involved in the regulation of gene and isoform expression. Its automated demultiplexing capability and integration of transcriptomic and chromatin accessibility data make it a valuable tool for unraveling the complex regulatory mechanisms underlying gene expression.

### Availability and future directions

With trackplot, it is possible to integrate multiple data sources from a wide variety of genomic assays and generate publication-ready plots. It allows users to visualize NGS data with flexible formats of input files as well as outputs. To ensure maximum reproducibility, Trackplot is distributed through PyPI, Bioconda, and Docker, allowing for easy installation and usage in different computational environments. Moreover, it also could be used via a command line or an API for an interactive environment such as Jupyter Notebook. In summary, trackplot offers an easy, fast, and reliable method for visualizing genomic data. The tool is written in Python and JavaScript which greatly facilitates future maintenance, and we will continue to maintain the updates and upgrades of the package based on suggestions and comments from the community.

The python package Trackplot is open-source and freely available on Docker (https://hub.docker.com/r/ygidtu/trackplot), GitHub (https://github.com/ygidtu/Trackplot), PyPI (https://pypi.org/project/Trackplot/) and Bioconda (https://anaconda.org/bioconda/trackplot). The script to generate S2–S4 Figs and several reproducible examples are available on GitHub (https://github.com/ygidtu/Trackplot/tree/main/example/Article_figures).

## Supporting information

S1 Fig We conducted a comparative analysis of runtime (A) and RAM usage (B) among ggsashimi, the sashimi function of MISO, and our tool using RNA-seq data obtained from https://www.ncbi.nlm.nih.gov/bioproject/PRJNA229103 (Wilcox test, ****: p < 10–5). Additionally, we assessed the time required for preparing config files for the sashimi function in MISO (C). For this evaluation, we randomly selected 1000 genes from the entire genome and tested the tools using 1, 5, 10, and 15 cores. Since ggsashimi lacked support for parallel processing, we conducted each test using only one core, resulting in four independent tests. Our results indicated that Trackplot outperformed ggsashimi in terms of runtime (A) and RAM usage for single core usage (B, upper left). However, MISO demonstrated faster and more efficient performance compared to the other tools. It is important to note that MISO generates multiple intermediate files for each job in its pipeline, resulting in lower peak memory usage but longer preprocessing times (C). Furthermore, MISO restricts users to generating plots exclusively based on the results obtained from MISO and limits the visualization to a fixed coordinate corresponding to a specific alternative splice event. This limitation hinders the flexibility to explore different regions of interest. As Trackplot offers a parallel processing option to expedite the handling of numerous files, such as expression QTL, it exhibited less efficient performance in terms of RAM usage (B) when multiple cores were utilized.
(TIF)

S2 Fig Integration of multiple NGS data source for revealing splicing regulation and outcomes. (A) Sashimi plot with protein functional description for the time-course RNA-seq data with biological replicates from TNP model during tumorigenesis. Tracks indicated RNA-seq density. The span junction line indicated the middle exon (highlight with dash line) was decreasing during tumorigenesis, and its splice percent in (*ψ*) was highlighted below label of each track. Bottom, the ENSEMBL gene annotation (black) and its protein domain information which was highlighted with pink background indicated different usage of isoforms with distinct functional domain during tumorigenesis. (B) Sashimi plot for combinatorial expression, splicing regulation and evolutionarily conserved score of *PTBP3*. Tracks indicated the signal from different format files as input, including PTBP1 eCLIP-seq (bigWig), KD-RNA-seq (BAM) and PhastCons score (bigWig). Tracks for eCLIP-seq indicated that *PTBP1* directly bind the PTBP3 exon 2 with highly conserved score (PhastCons track), and the exon 2 was significantly increased under perturbation of *PTBP1* (*PTBP1*_KD and *PTBP1*_WT tracks). Trackplot provides direct evidence that the alternative splice of PTBP3 exon 2 is likely regulated by PTBP1.
(TIF)

S3 Fig Visualization of full-length sequencing data. (A) Sashimi plot of cerebral organoid full-length sequencing data. Same data with different formats was present by conventional coverage plot and read-by-read style plot that each line represents an individual read, respectively. The box highlighted a mutually exclusive exons (MXE) event. Bottom, the ENSEMBL gene annotation (black) and its protein domain information which was highlighted with pink background indicated the different functional protein outcomes caused by MXE. (B) read-by-read style track plot of nanopore native RNA sequencing data. The red part at end of each read and the blue dot on each read represented the length of poly(A) and the status of m6A modification.
(TIF)

S4 Fig Decoding single-cell multiomics data. (A) Sashimi plot of 10x bone marrow datasets. Top track showed a density plot before demultiplexing, and tracks highlighted with pink background indicated strand-aware demultiplexing performed by Trackplot. The three peaks at each track represented three distinct isoforms and the polyadenylation site was indicated by blue dash line which was identified by SCAPE. Bottom, the ENSEMBL gene annotation of *Tubb5*. After strand-aware demultiplexing by Trackplot, it suggested that only pA1 and pA3 were generated by alteration of 3’ processing of the gene *Tubb5-201*, as we observed two sense peaks on 3’UTR of *Tubb5-201*. Surprisingly, the middle peak showed an opposite stand suggesting that pA2 was original from an anti-sense isoform of the *Tubb5-201*. (B) Integration of single-cell transcriptional and chromatin accessibility profiling. The tracks labeled with ATAC and Gene expression represented the signal from simultaneous profiling of ATAC and gene expression for a cell, respectively. The blue line indicated that there were two polyadenylation sites inferred by SCAPE. (C) The running time, sequencing depth, and the number of data tracks/categories were recorded during the generation of plots for the supplementary figures using Trackplot.
(TIF)
