# Supplementary figures and images for: Trackplot: A flexible toolkit for combinatorial analysis of genomic data
Source: PLoS Comput Biol. 2023 Sep 5;19(9):e1011477. doi: 10.1371/journal.pcbi.1011477 (PMC10503704; doi:10.1371/journal.pcbi.1011477)

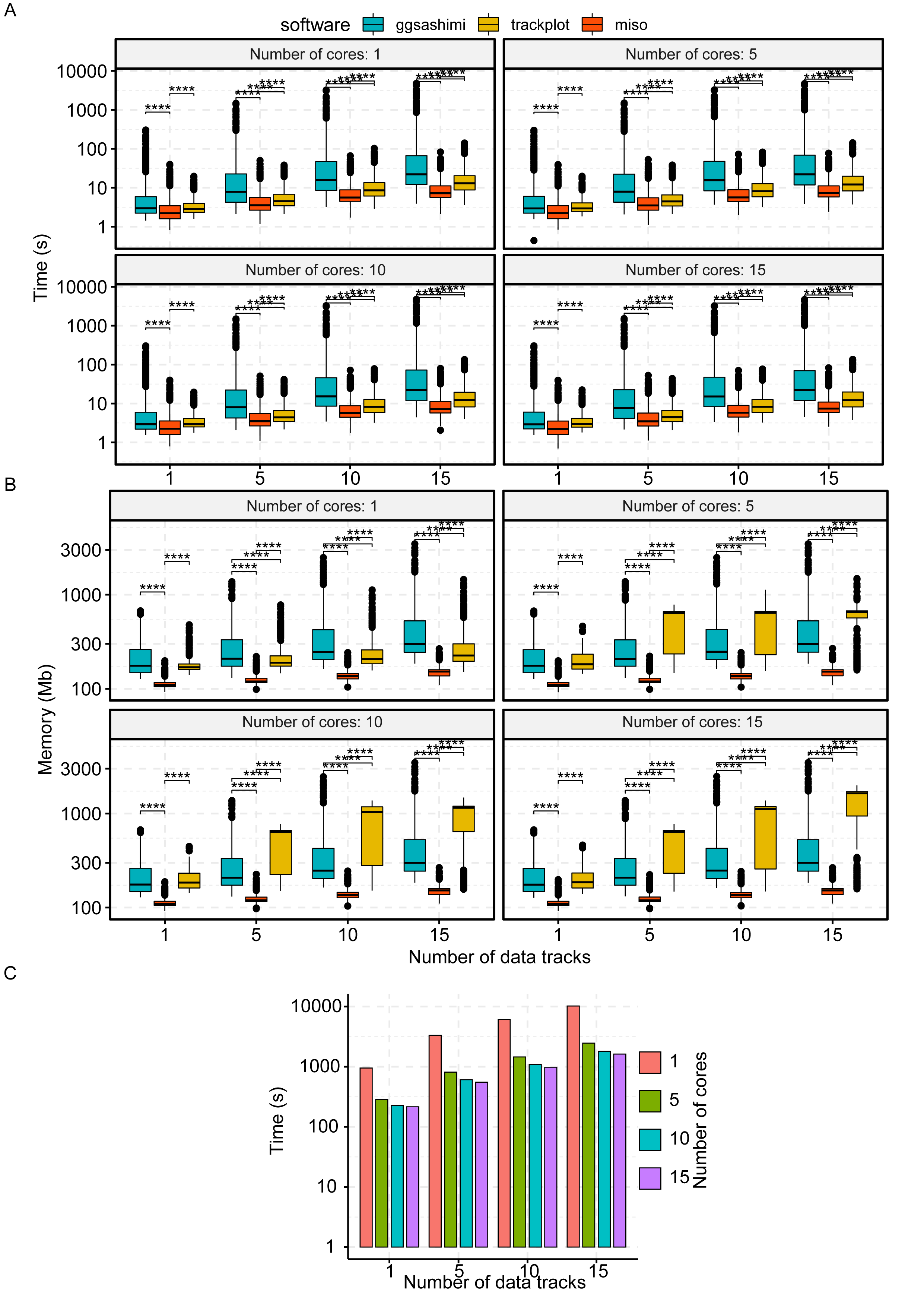

Supplement: S1 Fig — We conducted a comparative analysis of runtime (A) and RAM usage (B) among ggsashimi, the sashimi function of MISO, and our tool using RNA-seq data obtained from https://www.ncbi.nlm.nih.gov/bioproject/PRJNA229103 (Wilcox test, ****: p < 10–5). Additionally, we assessed the time required for preparing config files for the sashimi function in MISO (C). For this evaluation, we randomly selected 1000 genes from the entire genome and tested the tools using 1, 5, 10, and 15 cores. Since ggsashimi lacked support for parallel processing, we conducted each test using only one core, resulting in four independent tests. Our results indicated that Trackplot outperformed ggsashimi in terms of runtime (A) and RAM usage for single core usage (B, upper left). However, MISO demonstrated faster and more efficient performance compared to the other tools. It is important to note that MISO generates multiple intermediate files for each job in its pipeline, resulting in lower peak memory usage but longer preprocessing times (C). Furthermore, MISO restricts users to generating plots exclusively based on the results obtained from MISO and limits the visualization to a fixed coordinate corresponding to a specific alternative splice event. This limitation hinders the flexibility to explore different regions of interest. As Trackplot offers a parallel processing option to expedite the handling of numerous files, such as expression QTL, it exhibited less efficient performance in terms of RAM usage (B) when multiple cores were utilized. (TIF) [file pcbi.1011477.s001.tif]

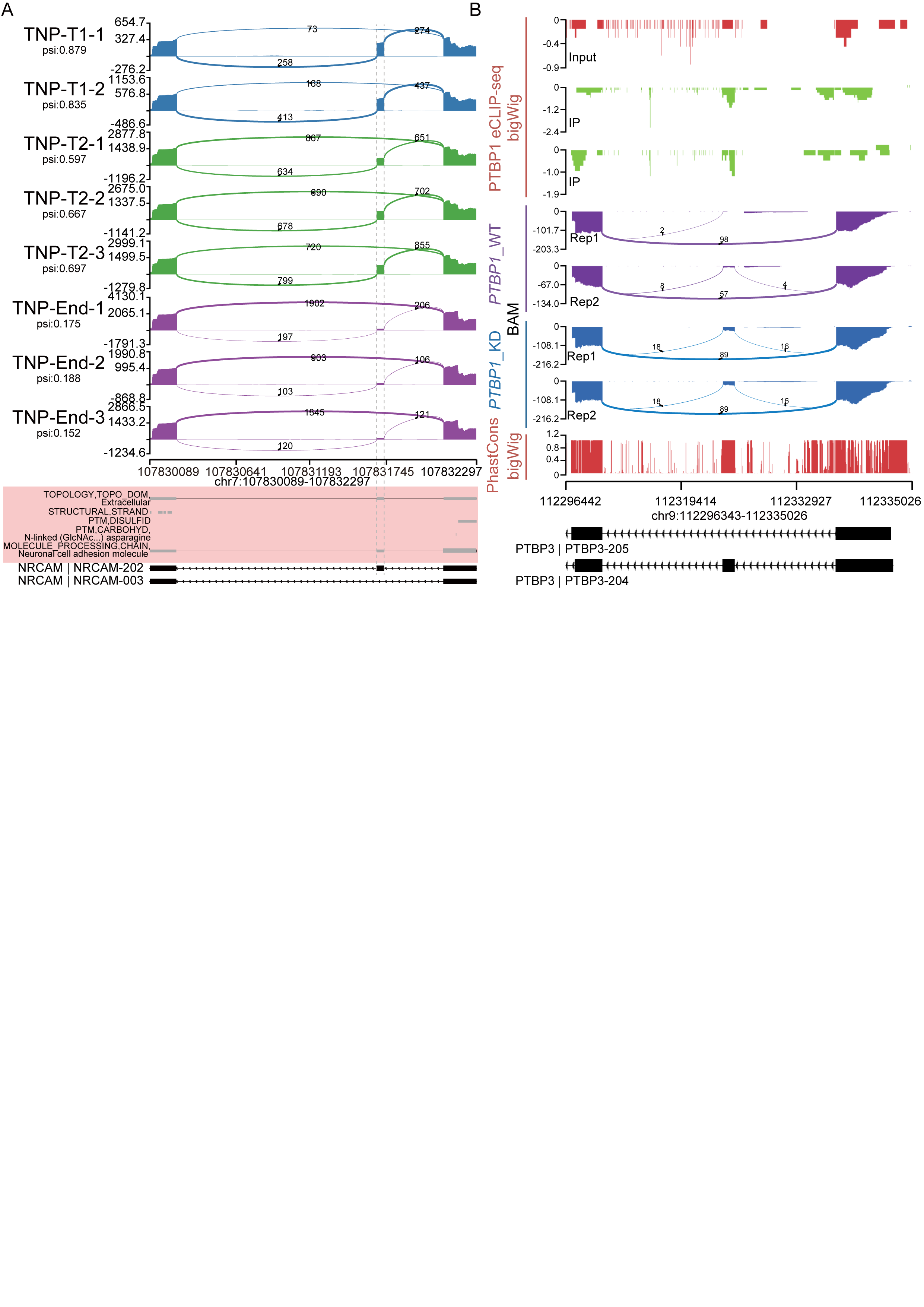

Supplement: S2 Fig — (A) Sashimi plot with protein functional description for the time-course RNA-seq data with biological replicates from TNP model during tumorigenesis. Tracks indicated RNA-seq density. The span junction line indicated the middle exon (highlight with dash line) was decreasing during tumorigenesis, and its splice percent in (ψ) was highlighted below label of each track. Bottom, the ENSEMBL gene annotation (black) and its protein domain information which was highlighted with pink background indicated different usage of isoforms with distinct functional domain during tumorigenesis. (B) Sashimi plot for combinatorial expression, splicing regulation and evolutionarily conserved score of PTBP3. Tracks indicated the signal from different format files as input, including PTBP1 eCLIP-seq (bigWig), KD-RNA-seq (BAM) and PhastCons score (bigWig). Tracks for eCLIP-seq indicated that PTBP1 directly bind the PTBP3 exon 2 with highly conserved score (PhastCons track), and the exon 2 was significantly increased under perturbation of PTBP1 (PTBP1_KD and PTBP1_WT tracks). Trackplot provides direct evidence that the alternative splice of PTBP3 exon 2 is likely regulated by PTBP1. (TIF) [file pcbi.1011477.s002.tif]

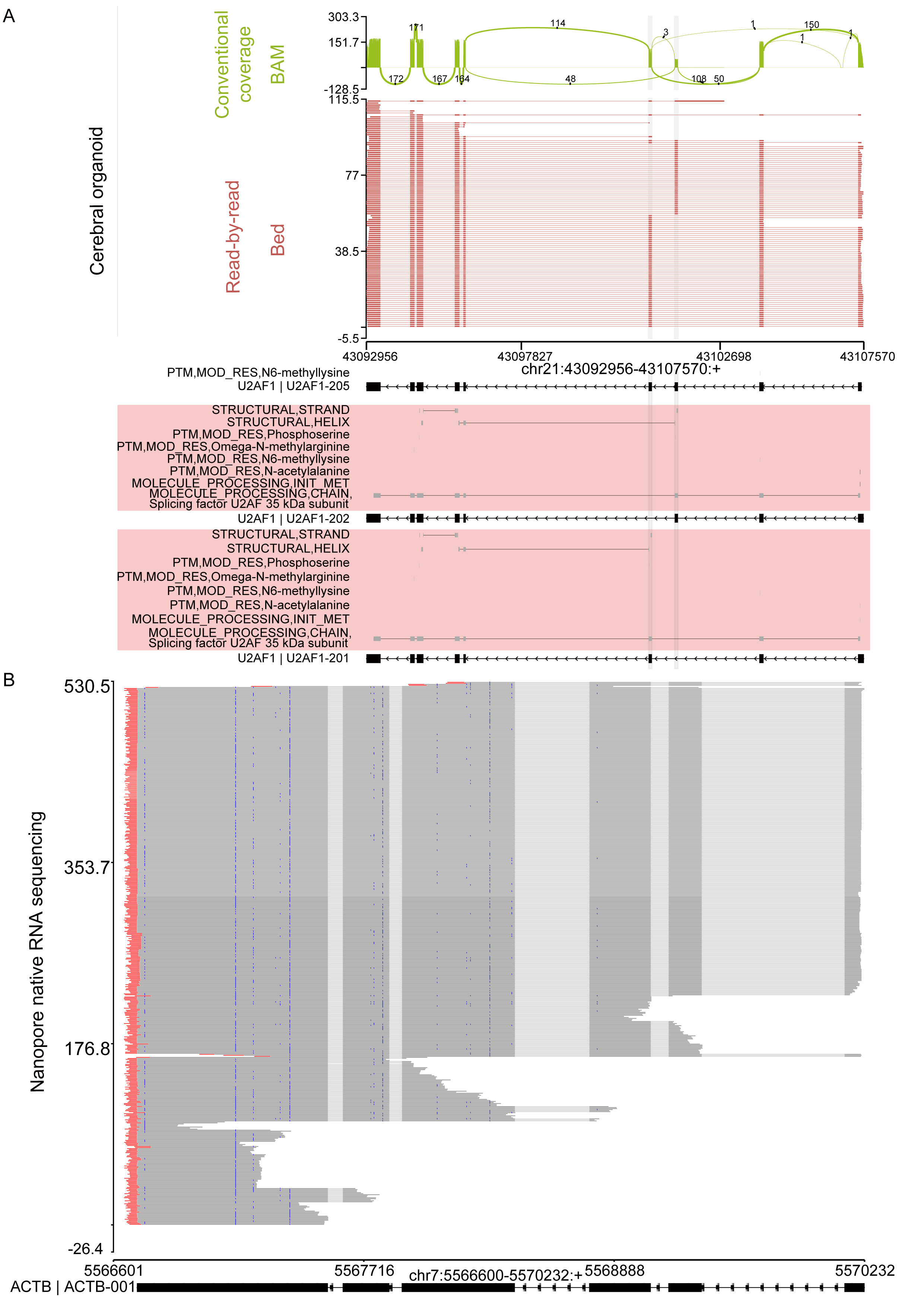

Supplement: S3 Fig — (A) Sashimi plot of cerebral organoid full-length sequencing data. Same data with different formats was present by conventional coverage plot and read-by-read style plot that each line represents an individual read, respectively. The box highlighted a mutually exclusive exons (MXE) event. Bottom, the ENSEMBL gene annotation (black) and its protein domain information which was highlighted with pink background indicated the different functional protein outcomes caused by MXE. (B) read-by-read style track plot of nanopore native RNA sequencing data. The red part at end of each read and the blue dot on each read represented the length of poly(A) and the status of m6A modification. (TIF) [file pcbi.1011477.s003.tif]

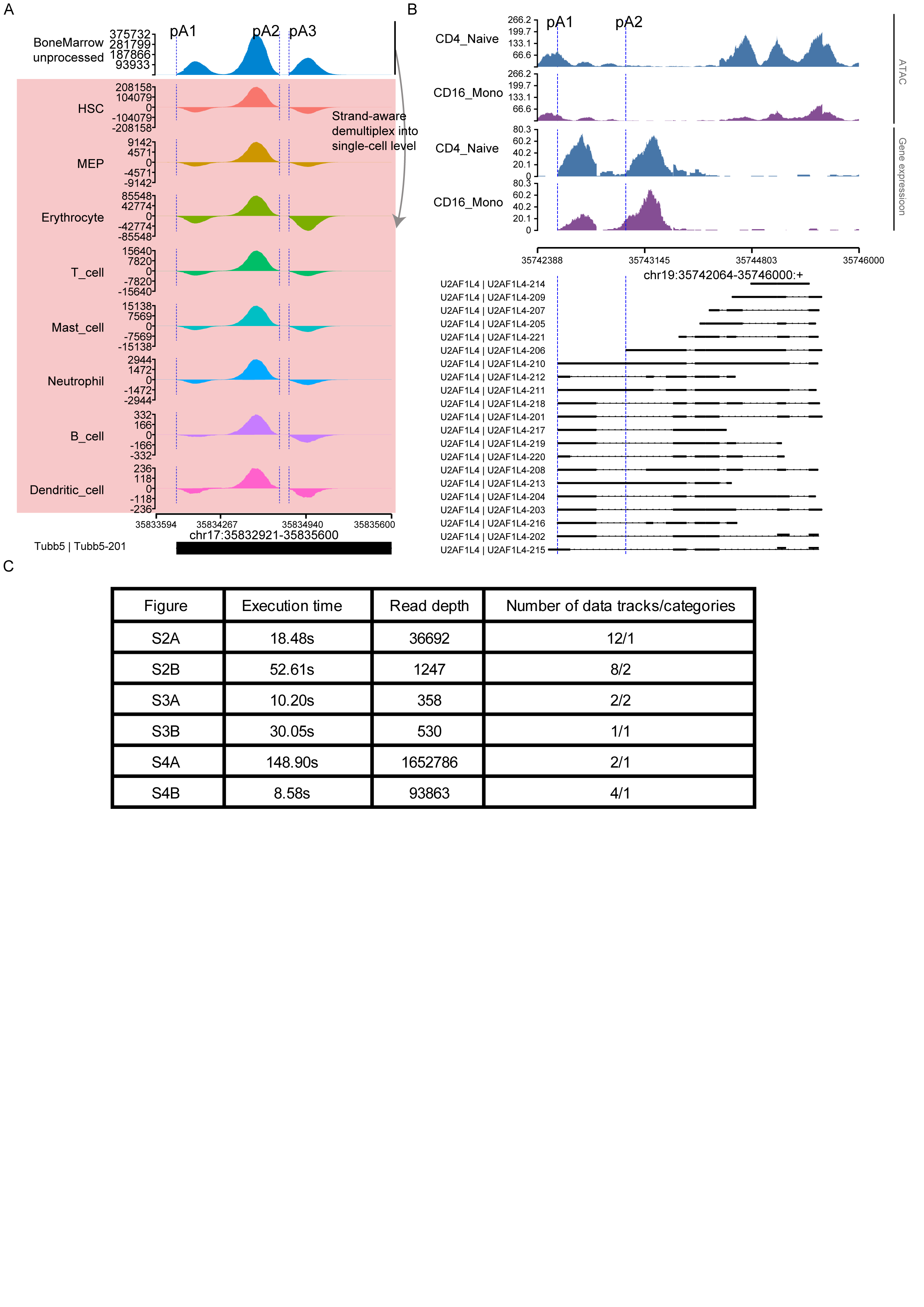

Supplement: S4 Fig — (A) Sashimi plot of 10x bone marrow datasets. Top track showed a density plot before demultiplexing, and tracks highlighted with pink background indicated strand-aware demultiplexing performed by Trackplot. The three peaks at each track represented three distinct isoforms and the polyadenylation site was indicated by blue dash line which was identified by SCAPE. Bottom, the ENSEMBL gene annotation of Tubb5. After strand-aware demultiplexing by Trackplot, it suggested that only pA1 and pA3 were generated by alteration of 3’ processing of the gene Tubb5-201, as we observed two sense peaks on 3’UTR of Tubb5-201. Surprisingly, the middle peak showed an opposite stand suggesting that pA2 was original from an anti-sense isoform of the Tubb5-201. (B) Integration of single-cell transcriptional and chromatin accessibility profiling. The tracks labeled with ATAC and Gene expression represented the signal from simultaneous profiling of ATAC and gene expression for a cell, respectively. The blue line indicated that there were two polyadenylation sites inferred by SCAPE. (C) The running time, sequencing depth, and the number of data tracks/categories were recorded during the generation of plots for the supplementary figures using Trackplot. (TIF) [file pcbi.1011477.s004.tif]
